# Growth in girls with Turner syndrome

**DOI:** 10.3389/fendo.2022.1068128

**Published:** 2023-01-12

**Authors:** Tsuyoshi Isojima, Susumu Yokoya

**Affiliations:** ^1^ Department of Pediatrics, Toranomon Hospital, Tokyo, Japan; ^2^ Department of Pediatrics, Teikyo University School of Medicine, Tokyo, Japan; ^3^ Fukushima Global Medical Science Center, Fukushima Medical University, Fukushima, Japan

**Keywords:** Turner syndrome, growth, growth chart, secular trend, growth hormone, estrogen

## Abstract

Turner syndrome (TS) is a chromosomal disorder affecting females characterized by short stature and gonadal dysgenesis. Untreated girls with TS reportedly are approximately 20-cm shorter than normal girls within their respective populations. The growth patterns of girls with TS also differ from those of the general population. They are born a little smaller than the normal population possibly due to a mild developmental delay in the uterus. After birth, their growth velocity declines sharply until 2 years of age, then continues to decline gradually until the pubertal age of normal children and then drops drastically around the pubertal period of normal children because of the lack of a pubertal spurt. After puberty, their growth velocity increases a little because of the lack of epiphyseal closure. A secular trend in height growth has been observed in girls with TS so growth in excess of the secular trend should be used wherever available in evaluating the growth in these girls. Growth hormone (GH) has been used to accelerate growth and is known to increase adult height. Estrogen replacement treatment is also necessary for most girls with TS because of hypergonadotropic hypogonadism. Therefore, both GH therapy and estrogen replacement treatment are essential in girls with TS. An optimal treatment should be determined considering both GH treatment and age-appropriate induction of puberty. In this review, we discuss the growth in girls with TS, including overall growth, pubertal growth, the secular trend, growth-promoting treatment, and sex hormone replacement treatment.

## 1 Introduction

Turner syndrome (TS) is a chromosomal disorder characterized by short stature and gonadal dysgenesis. TS affects 1 in 2,000–2,500 live female births and is the most common chromosomal disorder. Diagnosis is confirmed by chromosomal examination (G-band analysis) and is defined as having a karyotype that contains a cell line of monosomy lacking at least a distal major part in the short arm of the X chromosome (1–3). One of the most significant features of the syndrome is short stature. Untreated girls are reported to be approximately 20-cm shorter than normal girls within their respective populations (4). In addition to their short stature, the growth patterns of girls with TS differ from those of the general population mainly because of the short stature homeobox-containing gene on the X chromosome haploinsufficiency and their ovarian insufficiency. Growth hormone (GH) has been used to accelerate growth and is known to increase adult height (5).

Disease-specific growth charts have been developed for understanding growth patterns of individuals with TS and detecting their deviations from typical growth patterns (6, 7). These charts are very useful for evaluating the effects of growth-promoting treatment among patients as well. At present, disease-specific growth charts are available for several syndromes, such as TS, Prader–Willi syndrome, Down syndrome, Williams syndrome, and Noonan syndrome (8–14). TS-specific growth charts have been constructed in many countries worldwide (8, 9, 15–26) and are widely used for following up patients and evaluating the effect of growth-promoting treatment. In addition, TS-specific growth charts have had a role in revealing the overall growth patterns of girls with TS. We previously discussed our experiences in establishing disease-specific growth charts (7), and the main characteristics of several TS-specific growth charts are summarized in **Table 1**.

**Table 1:** The main characteristics of several TS-specific growth charts.

Growth chart	Characteristics	Country
Ranke M.B. et al., 1983 (15)	● Mixed longitudinal and cross-sectional data of 150 subjects in Germany were used for the establishment. ● Smoothing was not performed in the growth curves.	Germany
Ranke A.J. et al., 1985 (16)	● The combined statistics from three different countries including the data by Ranke M.B. et al., 1983 was computed for each age group. ● Only a height curve was presented.	Germany, Finland, France
Massa G. et al., 1990 (17)	● Mixed longitudinal and cross-sectional data of 100 subjects in Belgium were used for the establishment. ● Smoothing was not performed in the growth curves. ● Subjects with both spontaneous and induced puberty were included for the analyses.	Belgium
Haeusler G. et al., 1992 (18)	● Polynomial fitting equation was utilized for the establishment using mixed longitudinal and cross-sectional data of 141 subjects in Austria.	Austria
Bernasconi S. et al., 1994 (19)	● Mixed longitudinal and cross-sectional data of 772 subjects in Italy were used for the establishment. ● Subjects with both spontaneous and induced puberty were included for the analyses.	Italy
Sempé M. et al. 1996 (20)	● Computer modelling was performed for the establishment using the case records of 167 subjects in Lyon.	France
Rogen-Westerlaken C. et al., 1997 (21)	● TS-specific growth curves for Northern Europeans, the tallest people in the world. ● ICP function and regression equations were utilized for the establishment using mixed longitudinal and cross-sectional data of 598 subjects in Netherland, Denmark and Sweden.	Netherlands, Denmark, Sweden
Isojima T. et al., 2010 (9)	● TS-specific growth curves beyond the secular trend. ● The LMS method was utilized for the establishment using mixed longitudinal and cross-sectional data of 1,565 subjects in Japan.	Japan
El-Bassyouni H.T. et al., 2012 (22)	● Polynomial fitting equation was utilized for the establishment using the data of 93 subjects in Egypt. ● There is no information about the number of measurements in each age.	Egypt
Darendeliler F. et al., 2015 (23)	● The LMS method was utilized for the establishment using cross-sectional data of 842 subjects in Turkey.	Turkey
Khadilkar V.V. et al., 2020 (24)	● The cubic spline method and Lyon’s method were utilized for the establishment using mixed longitudinal and cross-sectional data of 113 subjects in western India.	India

In this review, we discuss the growth in girls with TS, including overall growth, pubertal growth, secular trend, growth-promoting treatment, and sex hormone replacement treatment.

## 1.1 Overall growth in girls with TS

**Figure 1:**
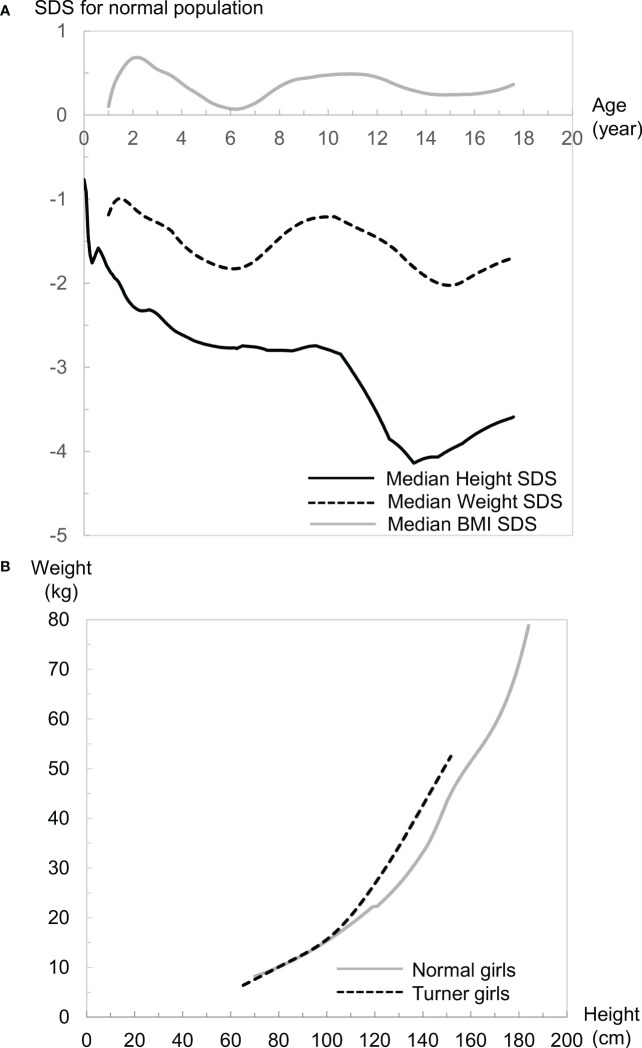
**(A)** Median height (black line), weight (black dotted line), and body mass index (BMI: gray line) standard deviation scores (SDS) of girls with Turner syndrome (TS) in the normal population. **(B)** Comparisons of weight for height (WFH) median lines between girls with TS (black dotted line) and girls of the normal population (gray line).

To visualize overall growth in girls with TS, median standard deviation scores (SDSs) of height, weight, body mass index (BMI), and weight for height (WFH) of Japanese girls with TS for Japanese normal population are shown in **Figure 1A** (height, weight, and BMI) and **Figure 1B** (WFH) (9, 27). The population of Japan is generally considered ethnically homogeneous and the growth patterns of girls with TS are similar among various ethnicities, judging from reported TS-specific growth charts (8, 9, 15–26). Therefore, we think that the visualization could help our understanding of overall growth in girls with TS not only in Japan but also in other countries.

Growth patterns of height in girls with TS differ from those of the normal population (**Figure 1A**). The median height SDS is smaller for Japanese girls with TS at birth (−0.76) than the median for the Japanese normal population, which is probably caused by a mild developmental delay in the uterus. This intrauterine growth retardation is also reported in several populations (15, 17–20). After birth, it declines sharply until 2 years of age. Then it continues to decline gradually until the pubertal age of normal children. Therefore, the height of most girls with TS is often within the normal range during infancy but usually falls below the fifth percentile of the general population ≤5 years of age (1). The height then drops drastically around the pubertal period of normal children because of the lack of pubertal spurt. This distinct growth pattern often leads to identification of growth problems during the period. After puberty, height recovers a little because of the lack of epiphyseal closure. These differences result in a median adult height SDS of −3.30 for Japanese girls with TS at age 20 years in a normal population, which is similar to overseas data (−2.54 to −4.15 SDS) (4). It is generally thought that untreated adults with TS are approximately 20-cm shorter than normal female adults (4), although ethnic background may affect adult height in girls with TS.

Girls with TS reportedly often become overweight as they grow up (15, 19, 21, 26), and many problems of females with TS in adult life are compounded by obesity (28). Weight-gain patterns in girls with TS are not as distinctive as height patterns, but are also different from normal-population weight-gain patterns, judging from median SDS changes during growth (**Figure 1A**). This weight-gain pattern declines between 1 and 6 years of age from −1.2 to −1.8 SDS, then increases to −1.2 SDS until 10 years of age. Subsequently, it gradually declines until 15 years of age, then increases again. When we evaluate weight gain in clinical settings, it is vital to consider the balance between height and weight gain, and the BMI and WFH are typically used for that purpose. Growth patterns of BMI in girls with TS differ from those in the normal population as well and resemble the weight-gain pattern except that the values are between 0 and 1 SDS and the increase after age 15 years can be negligible (**Figure 1A**). Considering the short stature in girls with TS, the positive value in BMI SDS should mean that girls with TS tend to be plump, which is consistent with the reported fact that girls with TS often become overweight as they grow up. WFH in girls with TS is almost the same as that in normal girls <100 cm tall, but more rapid increase in the WFH is evident in girls with TS ≥100 cm tall (**Figure 1B**). Considering that the median height at 6 years of age in Japanese girls with TS is 99.8 cm, the difference in body composition between girls with TS and the normal population might become evident from approximately 6 years of age. It is notable that the growth pattern of the median-weight SDS and -BMI SDS changes from a decrease to an increase at age 6 years when the BMI rebound occurs in the normal population on average. Accordingly, the growth patterns for BMI and WFH are totally different between girls with TS and the normal population, so careful attention is needed when assessing overweight or obesity status when employing the BMI and WFH as surrogate indices. We previously reported that there is a ≥30% discrepancy in the determination of overweight status in girls with TS aged ≥10 years when they were judged by two indices for the general population (29), although further investigation is essential to properly interpret this discrepancy.

### 1.2 Pubertal growth in girls with TS

Ovarian failure is one of the cardinal characteristics in girls with TS, but some of these girls undergo spontaneous puberty. It is unlikely that GH treatment affects the age of onset or the prevalence of spontaneous pubertal development in girls with TS (30, 31). Spontaneous thelarche has been reported in approximately one-third of girls with TS, which occurs most often in those with mosaicism, from many countries (17, 30–33), but only ≤6% of them have regular menstrual cycles, and spontaneous pregnancies are rare (30). Therefore, many girls with TS require sex hormone replacement therapy because of incomplete ovarian function (30, 31).

TS accompanied by spontaneous puberty should show a slightly different growth pattern from those without puberty. In fact, girls with TS and spontaneous puberty reportedly are significantly taller than those without spontaneous puberty at ≥12 years old, but the spontaneous pubertal development does not influence their adult height (17). Additionally, a slight premenarcheal growth spurt has been observed in girls with TS who experienced genital bleeding (16, 26). Therefore, when constructing TS-specific charts, two types of growth charts should be necessary during the pubertal period. However, for practical reasons, such as a limited number of pubertal subjects, only one type of TS-specific growth chart for girls without spontaneous puberty has been developed in many countries. It is vital for physicians to keep this fact in mind when they follow girls with TS and spontaneous puberty.

### 1.3 Secular trend of growth in girls with TS

**Figure 2:**
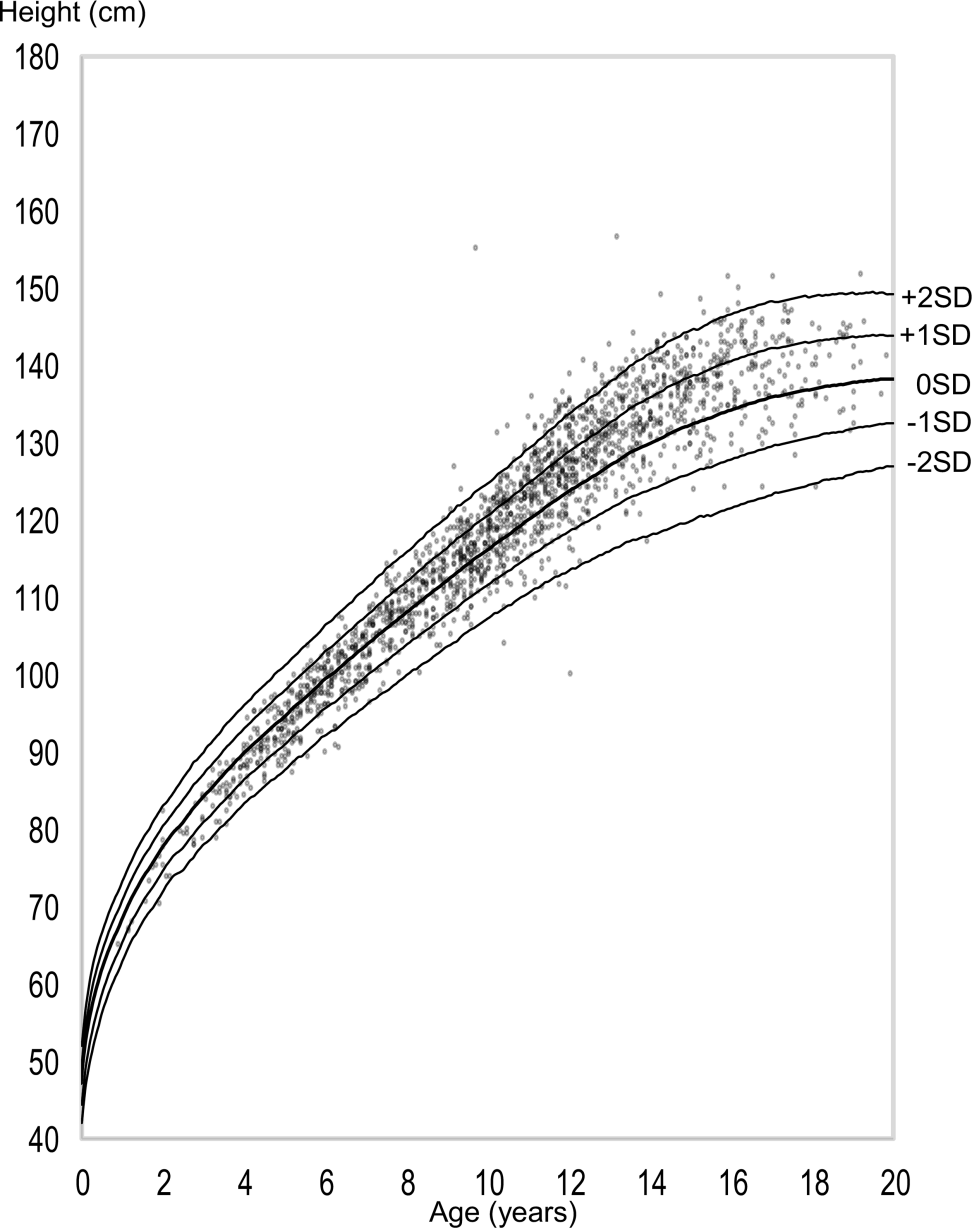
Scatter plots of Turner syndrome (TS) height data compiled by the Foundation of Growth Science, Japan, on the previous TS growth charts 26.

A secular trend of growth has been observed both in the normal population (34, 35) and girls with TS (8, 36). Regarding girls with TS, we previously reported the finding of a secular trend of height (8, 9). To investigate the secular trend of height in girls with TS in Japan, we plotted the data of 1,867 subjects compiled in the Foundation for Growth Science (FGS; a nationwide cohort of patients before and after GH treatment) from 1991 to 2004 for evaluation of eligibility for GH treatment on the previous TS-specific growth chart developed by Suwa in 1992 (26) (**Figure 2**). The data were distributed in the upper part of the growth charts in older ages, which we thought should be due to the secular trend of height. Considering that the previous TS-specific growth charts were reported in 1992 based on the data of 704 girls with TS (6,255 measurements) born from 1955 to 1989 (median: unknown) obtained from a questionnaire survey of hospitals, most of the subjects for the growth charts should have been born before the secular trend of height reached a plateau because the increase in height in Japan plateaued around 1990. Therefore, TS-specific charts should be updated, and we revised them for Japanese girls in 2010 using semi-longitudinal data (5,772 measurements) from both the FGS and two major hospitals for TS care. We found that the height at 20 years of age was 3.1-cm higher in the revised chart (141.3 cm) than in the previous chart (138.2 cm), which was nearly equivalent to the difference in mean female adult heights in the general population between 1970 (155.6 cm) and 1990 (157.9 cm) (8, 9). The secular trends of birth weight and height at the start of GH treatment in girls with TS also have been reported (36). Using anthropometric measurements in KIGS (Pfizer International Growth Database), girls with TS who were registered in the database between 1987 and 2012 and born between 1975 and 2004 were analyzed by comparing their data across 5-year birth-year groups. A significantly positive secular trend for birth weight (+0.18 SD) and height at the start of GH treatment (+0.38 SD) was detected in the study (36). In summary, there is a secular trend of growth in girls with TS, so growth references beyond the secular trend when evaluating growth should be used wherever available.

### 1.4 Growth-promoting treatment for girls with TS

GH has been shown to be effective for increasing adult height of 5–8 cm at a dosage of 42–50 μg/kg/day (5, 32, 37–41) and has been approved for growth-promoting treatment for girls with TS in many countries. On the other hand, a large interindividual variation in growth response to GH treatment has been reported (5, 32, 37–40), and the response of patients with TS was relatively poor and almost equal to that of achondroplasia and lower than that of GH deficiency or Noonan syndrome judging from the first year of GH treatment in clinical trials (32). In addition, several factors, such as younger age at initiation of GH treatment or timing of pubertal induction, affect treatment efficacy (5, 32, 37–40). To date, the optimal age for commencement of GH treatment has not been established, but ≥4 years of treatment prior to puberty has been associated with greater treatment effect (41). Evaluating the treatment effect is essential in clinical practice, and TS-specific growth charts are widely used for that purpose. An optimal treatment for girls with TS should be determined in each girl considering both GH treatment and an age-appropriate induction of puberty.

The existing literature shows that addition of oxandrolone, a nonaromatizable androgen (anabolic steroid), to GH has a beneficial effect on adult height in girls with TS (42, 43). Although the magnitude of the positive effect reportedly differs for different treatment regimens and subject characteristics (43), a recent systematic review showed that oxandrolone plus GH treatment was better than GH in terms of adult height by a mean difference of 2.7 cm (42). Oxandrolone is not available worldwide, so other anabolic steroids might be considered. For example, methenolone acetate is currently used in Japan (32). However, there is the potential for unwanted effects in a nonaromatizable androgen treatment, such as delayed breast development and dose-dependent virilization (including clitoromegaly, voice deepening, hirsutism, and acne). Therefore, caution is needed when administering this treatment. The consensus guideline from the 2016 Cincinnati International Turner Syndrome meeting suggested that concomitant treatment with oxandrolone from ≥10 years of age at 0.03 mg/kg/day and maintained at <0.05 mg/kg/day, if the diagnosis of TS and GH treatment initiation is delayed, and/or AH outcome is likely to be unsatisfactory with the standard GH dose alone (41).

### 1.5 Estrogen replacement treatment for girls with TS to increase growth

Estrogen replacement treatment is necessary for most girls with TS because of hypergonadotropic hypogonadism. Although the optimal procedure of the treatment has not yet been determined, the consensus guideline recommended that it should start between 11 and 12 years of age and then increase to adult dose over 2–3 years to mimic the physiology of healthy girls and improve psychological quality of life (41). Although the optimal regimen is still being elucidated, transdermal estradiol is preferable at this stage, because it is more physiological than other available drugs. On the other hand, estrogen replacement advances skeletal maturation and eventually causes epiphyseal fusion (32, 44, 45), which results in cessation of linear growth and lower adult height. For this reason, the timing of estrogen replacement induction has often been delayed until the mid-teen years, but it was recently reported that delaying pubertal induction beyond 12 years did not significantly improve adult height (43). Therefore, in recent clinical practice, a low dose of estrogen is thought to be preferable at treatment initiation, and a dose increase is usually determined by using TS-specific growth charts and evaluating each patient in terms of clinical symptoms, age, remaining growth potential, and patient satisfaction (41). Recently, ultra-low dose oral ethinyl estradiol initiation (25 ng/kg/day) during the prepubertal period combined with GH was reported to be effective for a modest synergistic increase in adult height (38, 46). In addition, there is the promising report that gradual increasing oral ethinyl estradiol from an extremely low dose of 1-5 ng/kg/day may produce good adult height (47). However, this treatment is not routinely recommended at this stage, because an optimal estrogen replacement treatment has not been determined and its long-term safety has not been assessed (41).

## 2 Concluding remarks

Girls with TS have short stature and hypergonadotropic hypogonadism, which should be treated in childhood. Both GH treatment and estrogen replacement therapy are essential in girls with TS. Since the growth pattern of these girls is very different from that of the normal population, understanding their growth pattern is vital for optimizing both their follow-up and treatment. TS-specific growth charts should be used for those purposes.

## Author contributions

TI conceived of the article, performed the literature search, and drafted the initial manuscript. TI and SY critically revised the work; both approve of the final submission. All authors contributed to the article and approved the submitted version.
